# mHealth Solutions for Perinatal Mental Health: Scoping Review and Appraisal Following the mHealth Index and Navigation Database Framework

**DOI:** 10.2196/30724

**Published:** 2022-01-17

**Authors:** Benedetta Spadaro, Nayra A Martin-Key, Erin Funnell, Sabine Bahn

**Affiliations:** 1 Cambridge Centre for Neuropsychiatric Research Department of Chemical Engineering and Biotechnology University of Cambridge Cambridge United Kingdom; 2 Psyomics Ltd Cambridge United Kingdom

**Keywords:** digital mental health, perinatal mental health, pregnancy, MIND, mobile phone

## Abstract

**Background:**

The ever-increasing pressure on health care systems has resulted in the underrecognition of perinatal mental disorders. Digital mental health tools such as apps could provide an option for accessible perinatal mental health screening and assessment. However, there is a lack of information regarding the availability and features of perinatal app options.

**Objective:**

This study aims to evaluate the current state of diagnostic and screening apps for perinatal mental health available on the Google Play Store (Android) and Apple App Store (iOS) and to review their features following the mHealth Index and Navigation Database framework.

**Methods:**

Following a scoping review approach, the Apple App Store and Google Play Store were systematically searched to identify perinatal mental health assessment apps. A total of 14 apps that met the inclusion criteria were downloaded and reviewed in a standardized manner using the mHealth Index and Navigation Database framework. The framework comprised 107 questions, allowing for a comprehensive assessment of app origin, functionality, engagement features, security, and clinical use.

**Results:**

Most apps were developed by for-profit companies (n=10), followed by private individuals (n=2) and trusted health care companies (n=2). Out of the 14 apps, 3 were available only on Android devices, 4 were available only on iOS devices, and 7 were available on both platforms. Approximately one-third of the apps (n=5) had been updated within the last 180 days. A total of 12 apps offered the Edinburgh Postnatal Depression Scale in its original version or in rephrased versions. Engagement, input, and output features included reminder notifications, connections to therapists, and free writing features. A total of 6 apps offered psychoeducational information and references. Privacy policies were available for 11 of the 14 apps, with a median Flesch-Kincaid reading grade level of 12.3. One app claimed to be compliant with the Health Insurance Portability and Accountability Act standards and 2 apps claimed to be compliant with General Data Protection Regulation. Of the apps that could be accessed in full (n=10), all appeared to fulfill the claims stated in their description. Only 1 app referenced a relevant peer-reviewed study. All the apps provided a warning for use, highlighting that the mental health assessment result should not be interpreted as a diagnosis or as a substitute for medical care. Only 3 apps allowed users to export or email their mental health test results.

**Conclusions:**

These results indicate that there are opportunities to improve perinatal mental health assessment apps. To this end, we recommend focusing on the development and validation of more comprehensive assessment tools, ensuring data protection and safety features are adequate for the intended app use, and improving data sharing features between users and health care professionals for timely support.

## Introduction

### Background

Perinatal mental health disorders are among the most common complications of childbearing. Recent systematic reviews have reported a prevalence of 17% for postnatal depression [1] and 15% to 20% and 10% for antenatal and postnatal anxiety disorders [2,3], respectively. These numbers are comparable with the prevalence rates of gestational diabetes (14.5%) [4] and preterm birth (11%) [5]. Critically, pregnancy can be a triggering event; 10% to 20% of pregnant individuals with bipolar disorder experience a relapse during or after pregnancy, often culminating in a severe mental illness episode (ie, postpartum psychosis and mania) requiring hospitalization [6-8]. In some individuals, postpartum psychosis can also be the first manifestation of bipolar disorder [9,10]. If left untreated, perinatal mental illnesses can lead to poorer maternal quality of life, emotional suffering, and an increased risk of suicide and infanticide. Mental health disorders throughout the perinatal period have also been found to diminish mother–infant bonding [11], impair breast feeding [12,13], and, in some cases, predict poor outcomes in social-emotional and cognitive development of children [11]*.* In addition to considerable maternal and infant morbidity, perinatal mental disorders carry substantial health and social care costs to society. For instance, in the United Kingdom alone, perinatal depression, anxiety, and psychosis cost approximately £8.10 (US $10.90) billion for each 1-year cohort of births, with £1.20 (US $1.60) billion falling directly on the National Health Service and social services [14]. In Australia, overall costs of perinatal mental illness were estimated to reach Aus $7.30 (US $ 5.30) billion, with Aus $643 (US $466) million loss in productivity and Aus $227 (US $164) million incurred in health costs within the first year of perinatal mental illness [15]. In the United States, it was projected that untreated perinatal mood and anxiety disorders costed US $14 billion for the 2017 birth cohort from conception to 5 years postpartum, with the average cost per affected mother–child averaging at US $31,800 [16].

The ever-increasing pressure on health care systems and lack of time and resources have resulted in a staggering underrecognition of postnatal depression and other perinatal disorders [17,18], with approximately 50% of cases of postnatal depression being undiagnosed [19]. This is likely because of an array of individual-level and organizational-level barriers, including negative attitudes and stigma regarding diagnosis; a lack of understanding of perinatal mental health disorders among pregnant individuals, their partners, and health care professionals; and fear of consequences [20]. Other challenges include cultural and language factors, resource fragmentation, and poor policy implementation [21].

In this regard, digital mental health services such as web-based assessments and apps could help alleviate some of the pressure put on in-person health care services and overcome barriers to help seeking, providing an alternative or complementary option for widespread perinatal mental health care provision. Indeed, mobile technology is rapidly expanding into the field of well-being and health care, with mobile health (mHealth) being among the fastest growing sectors with a compound annual growth rate of 32.5% [22]. Immediacy, accessibility, and affordability are among the potential benefits of using digital tools to identify perinatal mental health concerns. In the field of perinatal mental health, recent studies have highlighted that new mothers and those with postnatal depression are interested in using health apps [23], and recent studies have highlighted a growing research effort in the implementation of mHealth tools for psychoeducation and prevention of perinatal mental health concerns [24-26]. The COVID-19 pandemic has fueled interest in digital mental health solutions, as elevated levels of stress coupled with reduced in-person care prompted changes in mental health care provisions [26,27]. As a result, telemedicine services have been widely and successfully adopted in everyday perinatal care and mental health care [28-30] paving the way for the uptake of other digital health innovations such as apps.

### Objectives

Importantly, there still exists a large gap between interest in mental health digital tools [25] and a comprehensive understanding of the scientific integrity, clinical validity, and features of digital assessment tools for mental health [31-34]. In fact, 1 of the top 10 research priorities recently identified by the James Lind Alliance Priority Setting Partnership for digital technology in mental health care is to identify the best methods to evaluate and endorse mental health apps [35]. To this end, the objectives of this study are to identify apps that offer mental health screening or assessments for perinatal mental health available on the Google Play Store (Android) and the Apple App Store (iOS) and to review their features, including accessibility, privacy and security, clinical evidence, engagement style, and interoperability. Available apps were assessed using the mHealth Index and Navigation Database (MIND) framework [36,37]. The framework was developed by Lagan et al [36,37] in collaboration with the app evaluation model of the American Psychiatric Association (APA) [38], reflecting consensus from various stakeholders such as service users, social workers, psychiatrists, and data scientists to derive a ready-to-use resource for patients and clinicians alike [36,38,39]. The initial 38 open questions from the APA model served as the basis for the development of 107 questions that required binary (yes or no) or numeric responses covering app origin, functionality, security, engagement features, and clinical use. In the MIND framework, broad open questions from the APA model, for example, “What are the main engagement styles of the app?*”* were operationalized into 11 different types of engagement features. Similarly, the APA question “Is there a transparent privacy policy that is clear and accessible before use?” was operationalized into 2 objective questions: one regarding the presence of the privacy policy requiring a binary (yes or no) answer and the other prompting the rater to measure the reading level of the privacy policy (numeric response) to evaluate clarity. Thus, using defined and discrete evaluative questions, the MIND framework aims to be more objective and reproducible than the APA model.

The findings from this timely appraisal of perinatal diagnostic and screening apps have important implications for clinical practice and for the development of innovative ways to provide mental health care provisions throughout this complex time.

## Methods

### Overview

The objectives of this scoping review are to identify apps that offer perinatal mental health screening or assessments and to review their features against the MIND framework [36,37]. The scope comprised apps whose intended user populations specifically included adults in the perinatal period (ie, pregnant or had recently given birth) or health care professionals operating in perinatal health care. In consultation with a practicing psychiatrist (SB), interventions of interest included apps presenting questions and answers based digital screening and diagnostic tools completed by an individual or a health care professional on behalf of the individual, used for mental health screening, or as an aid in clinical decision-making.

We reported the review following the PRISMA-ScR (Preferred Reporting Items for Systematic Reviews and Meta-Analyses extension for Scoping Reviews) guidelines (Multimedia Appendix 1) [40].

### Search Strategy

Search terms to identify apps developed specifically for perinatal mental health were identified through a preliminary search of the Apple App Store and Google Play Store. Relevant synonyms and layperson alternatives were also included in the search. Layperson alternatives were included to capture app results as searched by consumers who may not use technical terminology. As a result, the following keyword combinations were used: *moms mental health*, *moms mental health screening*, *moms mental health assessment*, *mums mental health*, *mums mental health assessment*, *mums mental health screening*, *pregnancy mental health*, *maternal mental health*, *pregnancy mental health assessment*, *pregnancy mental health screening*, *perinatal*, *perinatal mental health*, *perinatal mental health assessment*, *perinatal mental health screening*, *postpartum*, *postpartum mental health*, *postpartum mental health assessment*, *mental health screening*, and *mental health assessment*.

These terms were used to search the 2 most widely used smartphone app stores, Apple App Store and Google Play Store, between January and February 2021, to identify publicly available apps.

### Inclusion Criteria

Apps were then shortlisted using the following inclusion criteria defined in consultation with a general adult psychiatrist (SB) and a practicing specialist perinatal psychiatrist:

1. Intended users of the app included at least 1 of the following groups: perinatal population and perinatal health care professionals.
2. The app offered a screening tool for any of the following conditions: bipolar disorder, major depressive disorder, postnatal depression, obsessive compulsive disorder, antenatal or postnatal anxiety, generalized anxiety disorder, agoraphobia, tokophobia, social phobia, panic disorder, insomnia, schizophrenia, (postpartum) psychosis, eating disorders (bulimia nervosa and anorexia nervosa), emotionally unstable personality disorder, alcohol abuse, substance abuse, (complex) posttraumatic stress disorder (PTSD), birth trauma, acute stress disorder, and adjustment disorder.
3. The app was available for download through the official Google Play or Apple App stores, and its description was available on the store.

### Exclusion Criteria

Apps that were not intended specifically for use by the perinatal population or health care professionals operating in the perinatal health field were excluded. Apps presenting screening or assessment tools designed solely for any type of health care professional training and examination preparation purposes were also excluded.

### Screening and App Selection

As the searches were performed on the Google Play Store and the Apple App Store, app names and links to the app stores were recorded on a Microsoft Excel spreadsheet. Duplicate apps retrieved using multiple search terms were then removed. A total of 2 independent reviewers (BS and NAMK) performed a blinded screening of the descriptions of all the identified apps. To decide whether the apps should be examined further, the independent reviewers assessed their eligibility against the inclusion criteria. Apps were labeled as *exclude*, *include*, or *maybe*. Any disagreements among the reviewers were discussed until a consensus was reached. The included apps were then analyzed and scored against the MIND framework [36].

### Assessment

Apps meeting the inclusion criteria were downloaded onto either a Pixel 5 (Android version 11) or an iPhone X (iOS version 14.2) for complete assessment. One reviewer (BS) transferred the MIND framework questions from the supplementary materials in Lagan et al into Microsoft Excel spreadsheets for data extraction and assessment of app features. Two independent reviewers (BS and NAMK) extracted the relevant information on separate spreadsheets. The 2 reviewers (BS and NAMK) independently performed the assessment of each app following the MIND framework as outlined by Lagan et al [36]. The extracted data and assessment results were then compared, and any discrepancies or disagreements were resolved by consensus discussion between those 2 authors.

The MIND framework comprised 107 questions on features related to the following app characteristics: (1) app origin including developer characteristics; (2) app functionality, which includes platform, number of downloads, average user-scored star rating, need for network connectivity, language, and price; (3) inputs and outputs, such as the presence of surveys, reminders, access to camera and microphone; (4) privacy and security features, including presence of an accessible privacy policy, data sharing policies and opt-outs, the presence of a crisis management feature; (5) evidence and clinical foundations, including adherence to app description claims, availability of evidence from feasibility studies, and compliance to clinical guidelines; (6) features and engagement style, which comprises characteristics such as the presence of tracking features, journaling, educational material, and peer support; (7) app use characteristics such as target audience and whether it is a self-managed tool or it is used together with a clinician; and (8) interoperability and data sharing matters, such as data ownership and interoperability with electronic medical records systems.

Reading level of the privacy policies of apps was calculated using an automatic text readability checker [41], as indicated in the MIND framework, resulting in a Flesh-Kincaid grade level [42], indicating a readability score corresponding to the US education grade level required for the reader to understand the text.

The MIND framework questions were designed to be answerable by any trained rater—clinician, peer, end user—and inform the identically titled public-facing database: the MIND [37,43]. The database was designed and implemented by the Division of Digital Psychiatry, a collaborative research group at the Beth Israel Deaconess Medical Center, a Harvard Medical School affiliate in Boston (MA, United States) that strives to create a comprehensive, easily searchable and updatable app database where apps are reviewed by trained raters following the MIND framework [36], and users can view app attributes and compare ratings. Hence, after performing the assessment of each of the apps following the MIND framework [36], we (BS and NAMK) searched the MIND database to compare our results with those of independent raters (Multimedia Appendix 2).

## Results

### Search Overview

**Figure 1:**
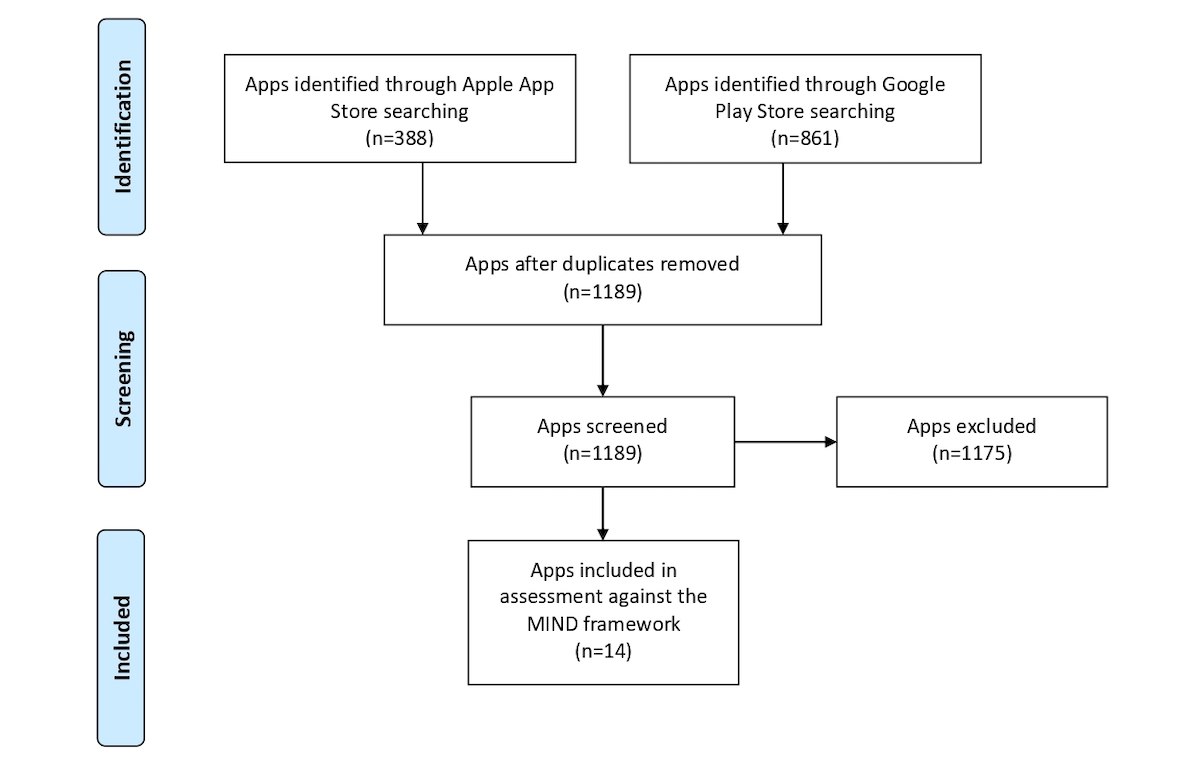
Flowchart of the search and selection strategy. MIND: mHealth Index and Navigation Database.

After removing all duplicates, a total of 1189 unique apps were identified, with 801 apps from the Google Play Store and 388 apps from the Apple App Store. Duplicate apps retrieved using multiple search terms were removed. After reviewing the description of the apps, a total of 1175 apps were excluded as they were of no relevance (Figure 1 and Table 1). A total of 14 apps were included for the analysis and scored against the MIND framework [36] (Multimedia Appendix 3). Overall, only 4 apps could be partially assessed on the basis of the information extracted from the app description on the Google Play Store and Apple App Store. Of these, 2 required a referral by a health care provider, and 2 apps repeatedly crashed; hence, the content could not be assessed.

**Table 1:** Excluded apps’ categories and frequencies.

App category	Category description	Frequencies,^a^ n (%)
Mental health screening and tracking (not perinatal)	Mental health screening apps not targeted to the perinatal population and mood tracking apps.	95 (7.99)
Mental health information and interventions	Apps offering mindfulness, meditation, journaling, and sleep help, apps offering mental health coaching (eg, panic attack management and suicide prevention) as well as cognitive behavioral therapy features, and apps offering mental-health information and psychoeducation.	399 (33.56)
Perinatal physical health and well-being	Apps offering information about physical changes occurring over pregnancy and postpartum as well as information on breastfeeding and well-being during the perinatal period.	102 (8.58)
Physical health information and tracking	Apps providing information on neonatal care and physical health, female and reproductive health, general physical health, and health tracking features (eg, heart rate).	99 (8.33)
Educational and reference material for health care professionals	Apps providing revision material for medical school and nursing exams as well as information for health care professionals on care pathways, and apps for scientific conferences and journals.	120 (10.09)
Telemedicine	Apps acting as platforms for video consultations, remote patient monitoring and care.	43 (3.62)
Fitness	Apps offering workouts and, in some cases, nutritional advice for pregnant individuals and mothers, and also fitness apps targeted to the general population.	147 (12.36)
Other	Miscellaneous apps eg, apps for audiobooks, music, shopping, traveling, games, brain training or logic games, cuisine, social media, and personality tests	170 (14.30)

^a^Total sample size includes all unique apps identified in the search (N=1189).

### App Origin and Functionality

Most apps were developed by for-profit providers (n=10) or private individuals (n=2), followed by trusted health care companies (n=2). Of the apps developed by trusted health care companies, 1 was developed in collaboration with an academic institution.

Of the 14 included apps, 3 were only available on Android devices, 4 were available only on Apple iOS devices, and 7 had versions for both platforms. Approximately one-third of the apps (n=5) had been updated within the last 180 days, with 11 apps updated within the last 13 months.

Most apps did not have sufficient reviews to display the average review rating. Of the apps that could be accessed, over half could work offline (n=7), not requiring an internet connection after downloading, and 2 apps could not function in the absence of an internet connection or required connectivity for some of their features. None of the apps presented accessibility features such as text size adjustment, a text-to-voice option, or color-blind color scheme options.

Examining costs, 8 apps were free, 1 was free to download, but it is unknown if it offered in-app purchases, 2 offered in-app purchases, or redirected the user to paid services for psychotherapy, whereas 2 apps were priced at US $0.99, and 1 was priced at US $1.09.

### Inputs and Outputs

All the apps offered self-assessment questionnaires to screen for perinatal depression. A total of 12 apps offered the Edinburgh Postnatal Depression Scale (EPDS) in its original version [44] or in slightly adapted versions. In addition to the EPDS, 1 app included the Patient Health Questionnaire–8-item scale [45], the Generalized Anxiety Disorder 7-item scale [46], the Insomnia Severity Index [47,48], the Perceived Stress Index [49], and questions about PTSD from the Mini-International Neuropsychiatric Interview [50] with attribution to original sources. A total of 3 apps offered the EPDS as well as additional tests not specifically targeted to the perinatal population such as tests for anxiety disorders, attention deficit hyperactivity disorder, PTSD, alcohol and substance use disorders, other various forms of addiction, eating disorders, personality disorders, bipolar disorder, and schizophrenia.

Most of the apps (n=8) provided the user with mental health screening scores and short summaries of their clinical significance. In addition, 2 apps provided a graphical depiction of the assessment results over time. A total of 3 apps offered the option of receiving reminder notifications to prompt the user to complete mental health screenings regularly.

Upon informing the user of their test scores, 8 apps recommended discussing results with a health care professional, 1 displayed the EPDS score without duty-of-care messaging, and the remaining 5 apps could not be viewed in full; therefore, it could not be determined whether users were advised to consult a health care professional. A total of 5 apps displayed duty-of-care messages and provided the user with crisis hotlines; in 4 cases, these were US-based hotlines and in 1 case, these were UK-based hotline; therefore, crisis advice was not personalized to the location of the user. Only 3 apps instantly displayed a duty-of-care message referring the user to clinical support and crisis hotlines if symptoms regarding suicidal ideation or self-harm were endorsed.

In addition to the screening tools, 2 apps presented a free writing, diary-like feature, and 1 app allowed the user to add notes after having taken the screening test. A total of 6 apps offered psychoeducation information or references to psychoeducational materials. An additional app presented psychoeducation videos that could not be viewed, and another app reported a link to psychoeducation information that was not functioning.

### Features and Engagement Style

Aside from screeners, assessments, reminders, and basic psychoeducation information, the apps did not include the great majority of engagement features listed in the MIND framework [36]. One app offered the user the possibility to connect with therapists online, whereas another app redirected users to BetterHelp, a web-based portal providing mental health services. One app allowed users to join a community on Facebook to share experiences and advice on mental health concerns. An additional app had a *safety plan* feature, allowing users to save motivational sentences and the details of up to 5 contacts to call in case of suicidal thoughts. None of the apps presented gamification features, such as gaining points or prizes for completing mental health assessments.

### Privacy and Security

Privacy policies were available either as a link from the app store description or in the app for 11 of the 14 apps. Overall, 1 app claimed to be compliant with the Health Insurance Portability and Accountability Act standards, and 2 additional apps claimed to be compliant with General Data Protection Regulation. Data use and purpose was declared in all the available privacy policies: in 2 cases data use was not detailed and in a further case, the information seemed to be related to the website of the developer and not specific to the app. A description of measures aimed at secure data collection and sharing was present in 6 of the 11 policies available. A total of 5 apps stated in their privacy policies that personal health information (PHI), including name, birthday, and mental health information would not leave the app, whereas PHI was shared in the other 6 apps. One-third of the apps in which PHI was shared did not report measures aimed at secure data collection and sharing. Deidentified data were shared by 7 apps, 5 of which also shared anonymized or aggregated user data. Only 2 apps of those that collected and shared data specifically stated in their privacy policies that the user could opt out of data collection. Three apps offered the option to delete all data related to the user upon request, whereas 2 apps allowed only for partial deletion of personal data. Out of the 11 privacy policies considered, 6 mentioned a crisis management feature (eg, a hotline number was included at the end of the mental health assessment). However, as discussed above, only 3 apps presented the user with an instant duty-of-care message upon presentation of suicidal ideation or self-harm. Finally, the median reading level of the privacy policies as measured using the Flesch-Kincaid reading grade level was 12.3, which corresponds to 12th grade, the final year of secondary school in the United States.

### Evidence, Clinical Foundations, Use, and Interoperability

Of the apps that could be accessed (n=10), all appeared to fulfill the claims stated in their descriptions. Importantly, the remaining 4 apps could not be evaluated on this criterion because they either crashed upon app launch or could not be accessed. All the assessed apps were patient-facing, with 3 of them being designed for use by both clinicians and patients.

One app referenced a relevant study conducted to test the app [51], where the usability of the app as a screening and management tool for perinatal depression was explored by gathering feedback from women in interviews. However, the study did not assess the efficacy of the diagnostic tool or psychoeducation content. One additional app referred to published, peer-reviewed studies, but it was unclear if the app tested in those studies corresponded to the current app version and the studies were not performed in perinatal populations.

All the apps provided a warning for use, highlighting that the mental health assessment result should not be interpreted as a diagnosis and that the app was not a substitute for medical care.

As a result, the apps were all regarded as *reference apps* and not *as self-help* tools. In the MIND framework, self-help apps are defined as providing activities that can be used for self-help and self-management, such as mood or symptom tracking or mindfulness exercises, whereas reference apps are defined as providing information and references but not necessarily activities. Although completing self-assessment questionnaires is a valuable activity, none of the apps that could be viewed in full offered activities that could help the user to manage the mental health concerns identified through the self-assessment activity.

The 3 apps allowed users to export or email their mental health test results. None of the apps seemed to have the necessary interoperability features to allow the sharing of app-gathered data to a medical record. The only app that offered an in-built connection with mental health therapists also allowed users to share their data with the therapist only after booking a therapy session.

## Discussion

### Principal Findings

Early mental health assessment strategies hold promise in supporting pregnant individuals and new mothers, but strategies vary widely among countries, with screening recommendations being subject to debates [52-54] and systematic reviews attempting to collect evidence to inform policy makers [55]. To date, access to mental health care is restricted to only a small proportion of pregnant individuals in need of mental health support [56]. During the COVID-19 pandemic, the increase in mental health concerns in the perinatal population has urged health care systems to expand care modalities [27,57]. As a result, guidelines compiled for perinatal care during the pandemic recommended asking pregnant individuals about their mental health at every antenatal and postnatal appointment and encouraged the use of digital means to deliver support [58].

Telemedicine has been the main digital tool used by health care professionals transitioning to remote care models, but apps have also started to be featured in mental health programs offered by health care providers and universities [59,60]. In a recent study conducted by our group, women, partners, and midwives expressed a strong interest in using a digital mental health assessment to screen, diagnose, and triage perinatal mental concerns [26]. This finding resonates with previous evidence showing that pregnant individuals are increasingly using digital tools as a source of health information during pregnancy and to enhance their understanding and involvement in pregnancy-related decision-making [61]. Moreover, studies also support the view that individuals are open to mental health discussions at perinatal visits and that delivery modality (eg, paper vs tablet) does not affect acceptability [62,63].

The interest in perinatal digital tools set a solid stepping stone for apps looking to meet the growing demand for accessible mental health screens and assessments. However, in contrast to this positive outlook, our results revealed an unsatisfactory landscape of existing app options for perinatal mental health screening and assessment. First, 14.3% (170/1189) of the results from the keyword search were completely unrelated to mental health and the perinatal period (*Other*; Table 1), which may be disheartening for users looking for help and relevant tools. Moreover, 12.36% (147/1189) of the excluded apps offered fitness programs, including workouts and weight loss (*Fitness*; Table 1), with such a focus on body image being potentially deleterious and triggering in the perinatal mental health context. A similar prevalence of apps targeting physical appearance and fitness was also reported by a recent systematic review specific to mHealth interventions for peripartum mood disorders [64].

In summary, of the included apps, several had not been recently updated, lacked accessibility features (eg, text size adjustments, text-to-voice options), and often presented functionality issues that could be deleterious and disheartening to users referring to the app for support. The screening tool most frequently encountered in this review was the EPDS. The EPDS can be used to screen for depression and anxiety [65]. However, most app descriptions and EPDS results reports focused heavily on perinatal depression. Only 1 app presented a more comprehensive screening pathway using questions and validated screening tools for depression, anxiety, insomnia, and PTSD symptoms. None of the apps acted as a diagnostic tool, instead they acted as screening tools. Indeed, the tools used by the apps such as the EPDS, Generalized Anxiety Disorder 7-item scale, Insomnia Severity Index, Patient Health Questionnaire–8-item scale, and Perceived Stress Index are screening tools aimed at identifying individuals who may benefit from further assessment. Screening tools are designed to have high sensitivity, whereas diagnostic tools are designed to have good content validity, test–retest reliability, good interrater reliability, and high specificity [66]. Using self-report inventories designed for screening purposes as a single means of deriving a diagnosis is inadequate and must be avoided. An in-depth interview with a clinician following the diagnostic criteria defined by the Diagnostic and Statistical Manual of Mental Disorders, fifth edition or the International Classification of Diseases, eleventh edition remains the gold standard for diagnostic assessment. Therefore, apps using self-reported inventories are far from being comprehensive enough to act as diagnostic tools, whereas they may be useful as a first-step screening tool that alerts individuals of the need for further assessment.

Indeed, if the screening result is positive, guiding app users toward a full diagnostic assessment is critical. Our review highlighted that, in some cases, the apps failed to provide clear, instant, and geographically relevant duty-of-care messages to users, even upon disclosure of self-harm or suicidality. Compliance with data security statutes and regulations (eg, Health Insurance Portability and Accountability Act and General Data Protection Regulation) has rarely been mentioned, and with a median Flesch-Kincaid reading grade level of 12.3, privacy policies were often above the suggested readability grade level (9-10) for the public [67]. Importantly, sharing of results with clinicians was enabled only by 3 apps, which allowed users to export or email their screening reports.

### Strengths and Limitations

The search strategy used to identify perinatal mental health assessment apps was comprehensive, and a completely blinded dual review process was used to ensure all relevant apps were included and to decrease the risk of reviewer bias.

The assessment was conducted using the MIND framework developed in collaboration with the APA [36]. In a recent review, the MIND framework operationalized by Lagan et al [37] showed significant overlap with other 70 app evaluation frameworks, highlighting its comprehensiveness and flexibility as a consolidated framework for app assessments. However, it should be noted that the framework does not include questions about ease of use, visual appeal, layout, and graphics. These are arguably subjective app features and are probably best assessed within the relevant clinical context and user population.

Critically, assessment of the apps strongly depended on the information disclosed by app developers in the app description, privacy policies, and functionality of the app itself. Our search did not include web or proprietary digital platforms that are not commercially available as apps on consumer-facing platforms. Recent literature shows that proprietary digital tools options exist and are offered by perinatal care providers [68], and there is also a disconnect between commercially available apps and academically available apps for perinatal mental health, which have been reviewed elsewhere [64].

Unlike electronic journal databases, app stores are not designed for systematic search and export of data [69]. For instance, there are challenges in removing duplicates directly in app store searches, and search results may be inconsistent given the nature of search algorithms and personalized app content of commercial app stores. Thus, it is challenging to replicate the search strategy reliably [70]. Currently, no guidelines exist for the conduct and reporting of systematic searches of app stores, but efforts are being made to reach a consensus [70].

Finally, our analysis aimed to analyze apps specifically designed and targeted to the perinatal population. However, our search revealed that there is a plethora of mental health screening, tracking, and interventions that do not specifically target the perinatal population (Table 1). The use of such tools may be helpful in identifying underlying mental health conditions but may not be able to capture dimensions that are specific to the perinatal period. Moreover, cutoffs, specificity, and sensitivity parameters of tools strongly depend on the population used for validation. Thus, the reliability of screening tools in a setting or population different from that in which the tool was developed cannot be guaranteed [71].

### Conclusions

This review of apps for perinatal mental health assessment following the MIND framework supports the view that there are gaps in the current app space. As a result, we recommend 3 areas of focus for app developers and clinicians in designing and evaluating apps for perinatal mental health assessments.

First, development of more comprehensive digital screening tools is required. Critically, although the identification of perinatal depression is facilitated by validated questionnaires such as the EPDS, there is less consensus on screening tools for other disorders with significant prevalence, including perinatal anxiety and substance use disorder. Systematic reviews of mental health screening tools [72-74] may be used to inform the development and assessment of digital questionnaires that aim to provide a more comprehensive screening. Formal validation against a gold standard is then required to establish the reliability and accuracy parameters of screening tools in the setting and population of interest.

Second, the importance of safety features cannot be overstated. Any developer aiming to design a mental health screening tool should be responsible for keeping PHI safe and providing adequate information about sources of help in case of disclosure of self-harm or suicidality. To this end, following data security statutes and keeping apps up-to-date with geographically relevant sources of help and crisis hotlines represent the very first steps toward a more secure and responsible development of mental health apps.

Third, app developers and clinicians should strive to increase interoperability and data sharing. Digital self-reported screening tools can increase access to mental health support and aid triage only if the user is encouraged to share their screening results with a health care professional. To this end, data sharing must be easy for users and clinicians alike. With only 3 apps of the reviewed apps allowing to export screening results in some form (email or PDF), we believe there exists an opportunity to develop tools that better integrate with current medical records systems. Enhancing data sharing in a secure manner is likely to increase the use of mental health screening apps and contribute to a better therapeutic alliance between app users, developers, and clinicians.

## Appendix

Multimedia Appendix 1 PRISMA-ScR (Preferred Reporting Items for Systematic Reviews and Meta-Analyses extension for Scoping Reviews) checklist.

Multimedia Appendix 2 Comparison of authors’ assessment with ratings available in the mHealth Index and Navigation Database sorted by rating category (platform, developer type, etc).

Multimedia Appendix 3 Results of the assessment of the included apps (n=14) following the questions (n=107) of the mHealth Index and Navigation Database framework.

## Abbreviations

APA: American Psychiatric Association
EPDS: Edinburgh Postnatal Depression Scale
MIND: mHealth Index and Navigation Database
PHI: personal health information
PRISMA-ScR: Preferred Reporting Items for Systematic Reviews and Meta-Analyses extension for Scoping Reviews
PTSD: posttraumatic stress disorder
