# Supplementary material for: mHealth Solutions for Perinatal Mental Health: Scoping Review and Appraisal Following the mHealth Index and Navigation Database Framework
Source: JMIR Mhealth Uhealth. 2022 Jan 17;10(1):e30724. doi: 10.2196/30724 (PMC8804959; doi:10.2196/30724)
Supplement: Multimedia Appendix 2 [file mhealth_v10i1e30724_app2.doc]

**Supplementary Table 2.** Comparison of authors’ assessment with ratings available in the MIND sorted by rating category (platform, developer type, etc.).

Only two apps, MGHPDS and Mental Health Tests, were present in the MIND, hence we compared our assessment results with the database ratings. We annotated differences and provided comments. The discrepancies that were encountered may suggest that app versions may differ between countries. Other discrepancies were due to differences in the rating categories included in the MIND.

| App Name  App Features | MGHPDS | | Comments | Mental Health Tests | | Comments |
| --- | --- | --- | --- | --- | --- | --- |
|  | MIND raters^1^ | Authors |  | MIND raters^2^ | Authors |  |
| **Platform** |  |  |  |  |  |  |
| Android | ✓ | ✓ |  | ✓ | ✓ |  |
| iOS | ✓ | ✓ |  | ✓ | ✓ |  |
| **Developer type** |  |  |  |  |  |  |
| Government | ✓ | ✓ |  |  |  |  |
| For Profit |  |  |  | ✓ | ✓ |  |
| Non-Profit | ✓ | ✓ |  |  |  |  |
| Healthcare | ✓ | ✓ |  |  |  |  |
| Academic |  |  |  |  |  |  |
| **Cost** |  |  |  |  |  |  |
| Free to Download | ✓ | ✓ |  | ✓ | ✓ |  |
| Totally Free | ✓ | ✓ |  | ✓ | ✓ |  |
| Payment |  |  |  |  |  |  |
| In-App Purchase |  |  |  |  |  |  |
| Subscription |  |  |  |  |  |  |
| **Access** |  |  |  |  |  |  |
| Spanish |  |  |  |  |  |  |
| Offline |  |  |  | ✓ | ✓ |  |
| Accessibility | ✓ |  | No text/audio adjustment feature was found in the version of the app analyzed by the authors. | ✓ |  | No text/audio adjustment feature was found in the version of the app analyzed by the authors |
| Own Data | ✓ |  | Details on data ownership were not stated in the full participation consent information nor in the privacy policy. |  |  |  |
| Export Data |  |  |  | ✓ | ✓ |  |
| Send Record | ✓ |  | The option to send a record was not found in the version of the app analyzed by the authors |  |  |  |
| **Privacies** |  |  |  |  |  |  |
| Has Policy | ✓ | ✓ |  | ✓ | ✓ |  |
| Device Storage |  |  |  |  |  |  |
| Server Storage | ✓ | ✓ |  | ✓ | ✓ |  |
| Can Delete Data | ✓ |  | Not specifically stated in the full participation consent information nor in the privacy policy. | ✓ | ✓ |  |
| Declares Purpose | ✓ | ✓ |  | ✓ | ✓ |  |
| Security Measures | ✓ | ✓ |  | ✓ | ✓ |  |
| PHI Shared |  |  |  | ✓ | ✓ |  |
| De-Indentifed Data Shared |  |  |  | ✓ | ✓ |  |
| Anonymized Data Shared | ✓ | ✓ |  | ✓ | ✓ |  |
| Data Collection Opt Out | ✓ | ✓ |  |  |  |  |
| Meets HIPAA | ✓ | ✓ |  |  |  |  |
| Crisis Management Features |  |  |  | ✓ | ✓ |  |
| **Clinical Foundations** |  |  |  |  |  |  |
| Well Written | ✓ |  | This question was not present in the MIND framework by Lagan et al. | ✓ |  | This question was not present in the MIND framework by Lagan et al. |
| Does as Claims | ✓ | ✓ |  | ✓ | ✓ |  |
| Patient Facing | ✓ | ✓ |  | ✓ | ✓ |  |
| Can Cause Harm |  |  |  |  |  |  |
| Use Warning | ✓ | ✓ |  | ✓ | ✓ |  |
| Supporting Studies |  |  |  |  |  |  |
| **Features** |  |  |  |  |  |  |
| Track Mood | ✓ | ✓ |  |  | ✓ | The app allows users to answer questions on their mood and track their test score over time. |
| Track Medication |  |  |  |  |  |  |
| Track Sleep |  |  |  |  |  |  |
| Track Symptoms | ✓ | ✓ |  | ✓ | ✓ |  |
| Productivity |  |  |  |  |  |  |
| Physical Health |  |  |  |  |  |  |
| Psychoeducation |  |  |  |  |  |  |
| Journaling | ✓ |  | No journaling feature was found in the version of the app analyzed by the authors. |  |  |  |
| Mindfulness |  |  |  |  |  |  |
| Deep Breathing |  |  |  |  |  |  |
| Picture Gallery/Hope Board |  |  |  |  |  |  |
| iCBT or Sleep Therapy |  |  |  |  |  |  |
| CBT |  |  |  |  |  |  |
| ACT |  |  |  |  |  |  |
| DBT |  |  |  |  |  |  |
| Peer Support |  |  |  |  |  |  |
| Coach/Therapist Connection |  |  |  |  |  |  |
| Biodata |  |  |  |  |  |  |
| Goal Setting/Habits | ✓ |  | No goal-setting feature was found in the version of the app analyzed by the authors. |  |  |  |
| Physical Health Exercises |  |  |  |  |  |  |
| Chatbot Interaction |  |  |  | ✓ | ✓ |  |
| **Supported Conditions** |  |  |  |  |  |  |
| Mood Disorders | ✓ |  | This question was not present in the MIND framework by Lagan et al. | ✓ |  | This question was not present in the MIND framework by Lagan et al. |
| Stress & Anxiety | ✓ |  |  |  |  |  |
| Sleep |  |  |  |  |  |  |
| Phobias |  |  |  |  |  |  |
| OCD |  |  |  | ✓ |  |  |
| Schizophrenia |  |  |  |  |  |  |
| Eating Disorders |  |  |  |  |  |  |
| Personality Disorders |  |  |  |  |  |  |
| Self-Harm |  |  |  |  |  |  |
| PTSD |  |  |  | ✓ |  |  |
| Substance Use |  |  |  | ✓ |  |  |
| **Engagements** |  |  |  |  |  |  |
| User Generated Data | ✓ | ✓ |  | ✓ | ✓ |  |
| Chat/Message |  |  |  |  |  |  |
| Assessments/Screenings | ✓ | ✓ |  | ✓ | ✓ |  |
| Real Time Response |  |  |  |  |  |  |
| Asynchronous Response |  |  |  |  |  |  |
| Gamification |  |  |  |  |  |  |
| Videos |  |  |  |  |  |  |
| Audio/Music/Scripts |  |  |  |  |  |  |
| AI Support |  |  |  |  |  |  |
| Peer Support |  |  |  |  |  |  |
| Network Support |  |  |  |  |  |  |
| Collaboration | ✓ |  | No medical care or advice is given in the application as stated in the consent form. "*The information is collected for research purposes only"*. |  |  |  |
| **Inputs** |  |  |  |  |  |  |
| Surveys | ✓ | ✓ |  | ✓ | ✓ |  |
| Diary | ✓ |  | No diary feature was found in the version of the app analyzed by the authors |  |  |  |
| Geolocation |  |  |  | ✓ | ✓ |  |
| Contact List |  |  |  |  |  |  |
| Camera |  |  |  |  |  |  |
| Microphone |  |  |  |  |  |  |
| Step Count |  |  |  |  |  |  |
| External Devices |  |  |  |  |  |  |
| Social Networks |  |  |  |  |  |  |
| **Outputs** |  |  |  |  |  |  |
| Notifications | ✓ | ✓ |  | ✓ | ✓ |  |
| References/Information | ✓ | ✓ |  |  | ✓ | The user can access information about the condition screened by the test by clicking the 'about' button after selecting the relevant test. |
| Social Network |  |  |  |  | ✓ | The user can share the link to the app through social networks via the 'share' option in the app menu. |
| Reminders |  |  |  |  |  |  |
| Graphs of Data |  |  |  |  | ✓ | The app offers the user a graphical representation of score of past tests. |
| Summary of Data |  |  |  | ✓ | ✓ |  |
| Link to Formal Care/Coaching |  |  |  |  | ✓ | The app provides the user with the link to BetterHelp and their mental healthcare services. |
| **Uses** |  |  |  |  |  |  |
| Self Help | ✓ |  | The authors regarded the app content as insufficient to allow the user to self-manage their concerns. |  |  |  |
| Reference | ✓ | ✓ |  | ✓ | ✓ |  |
| Hybrid |  |  |  |  |  |  |

***Key.*** Green ticked boxes indicate agreement between authors’ review and MIND reviews. Yellow boxes indicate differences between the app library and the authors review of app features. ^1^review of the 30^th^ March 2021, ^2^review of 12th April 2021.
